# Recent Advances in the Transition-Metal-Free Synthesis of Quinazolines

**DOI:** 10.3390/molecules28073227

**Published:** 2023-04-04

**Authors:** Rekha Tamatam, Dongyun Shin

**Affiliations:** 1 College of Pharmacy, Gachon University, 191 Hambakmoe-ro, Yeonsu-gu, Incheon 21936, Republic of Korea; 2 Gachon Pharmaceutical Research Institute, Gachon University, 191 Hambakmoe-ro, Yeonsu-gu, Incheon 21936, Republic of Korea

**Keywords:** quinazolines, transition-metal-free, synthesis, advantages, limitations, mechanism

## Abstract

Quinazolines are a privileged class of nitrogen-containing heterocycles, widely present in a variety of natural products and synthetic chemicals with a broad spectrum of biological and medicinal activities. Owing to their pharmaceutical applications and promising biological value, a variety of synthetic methodologies have been reported for these scaffolds. From the perspective of green and sustainable chemistry, transition-metal-free synthesis provides an alternative method for accessing several biologically active heterocycles. In this review, we summarize the recent progress achieved in the transition-metal-free synthesis of quinazolines and we cover the literature from 2015 to 2022. This aspect is present alongside the advantages, limitations, mechanistic rationalization, and future perspectives associated with the synthetic methodologies.

## 1. Introduction

**Figure 1:**
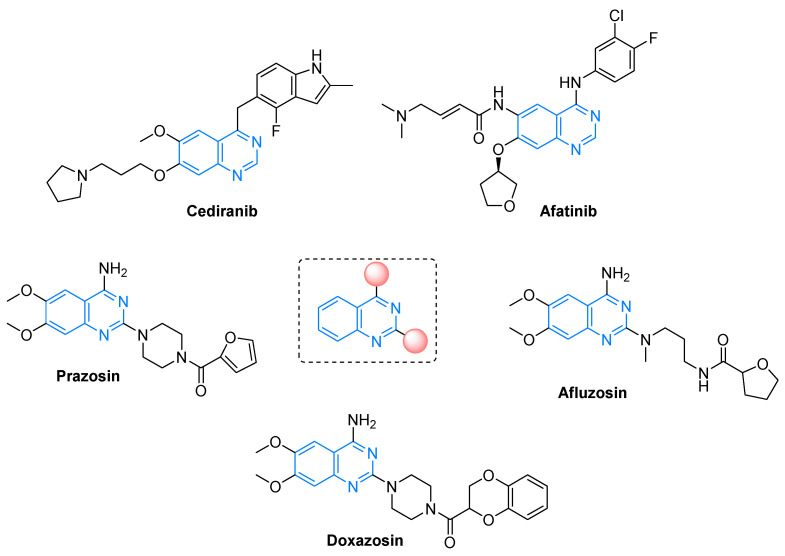
Structure of quinazoline-based drugs.

Nitrogen heterocycles are found in a large number of biologically active natural products as well as synthetic compounds [1,2]. Amongst them, quinazolines are an attractive class of heterocycles and serve as building blocks in making advanced intermediates and active pharmaceutical ingredients (APIs) for the pharmaceutical industry [3,4]. They have been found to possess a diverse range of pharmacological properties including anti-inflammatory [5], anticancer [6], anticonvulsant [7], anti-hypertensive [8], anti-HIV [9], anti-tubercular [10], antibacterial [11], antifungal [12], antiviral [13], antimalarial [14], anti-plasmodial [15], α-adrenergic blocker [16], antioxidant [17], and antitumor activities [18]. Additionally, quinazoline derivatives have been applied as photochemotherapeutic agents [19], JAK2, DNA-gyrase, PDE5, and EGFR tyrosine kinase inhibitors [20,21,22,23], and CB2 receptor agonists [24]. Quinazolines have been used in regimens for a wide range of physical ailments such as hematological cancer, liver metastasis (Cediranib), breast cancer (Afatinib), high blood sugar and hypertension (Prazosin), benign prostatic hyperplasia (Afluzosin), and prostate enlargement and high blood pressure (Doxazosin) [25,26,27,28]. These fused N-heterocyclic cores are present in lung cancer drugs and therapeutic agents for pneumonia and post-traumatic stress or anxiety disorders [29,30,31,32]. Some quinazoline-based drugs are illustrated in Figure 1.

**Figure 2:**
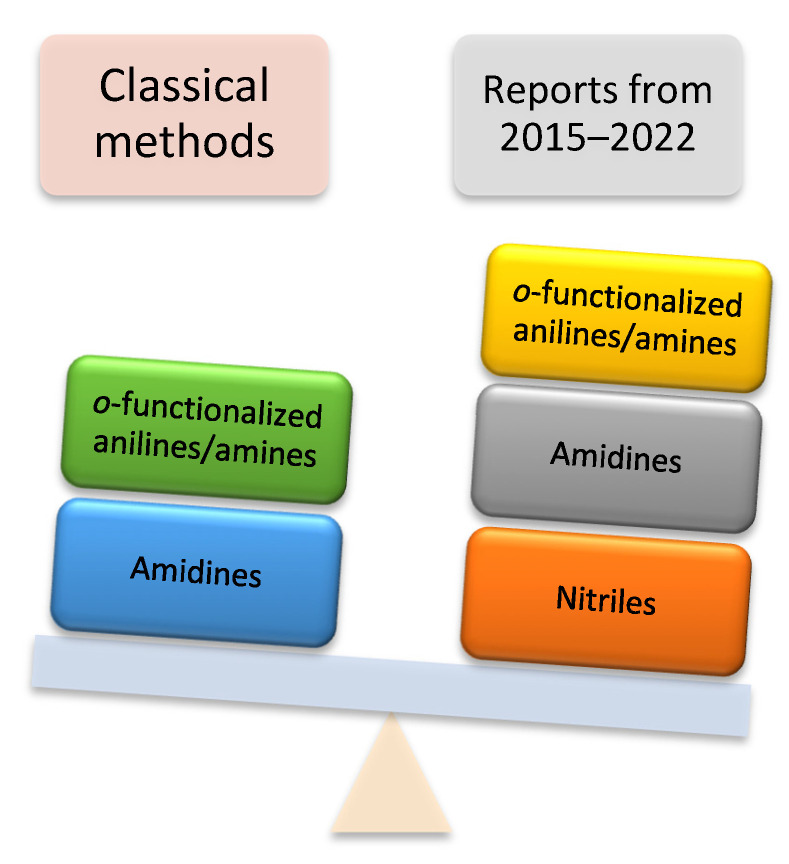
Substrates scope in classical methods vs. reports between 2015–2022.

Moreover, quinazolines are employed as fragments in the functionalized materials [33] and also serve as useful intermediates in fine chemicals [34]. Owing to this biologically active legacy, quinazolines have emerged as synthetic targets in the last few decades. The first example of the synthesis of quinazolines from cyanogen and anthranilic acid was reported by Griess in 1869 [35]. Consequently, several protocols have been developed since then [36,37,38,39]. Recently, transition-metal-free reactions have received considerable attention and are recognized as an indispensable tool in organic synthesis, as they are not only cost-effective but also environmentally friendly. The metal-free methodology compliments metal-mediated reactions by eliminating expensive metal complexes and toxic metal contamination [40]. There are two classical strategies for the construction of the quinazoline skeleton under metal-free conditions: (i) substituted quinazolines can be prepared from easily available ortho-functionalized anilines such as 2-aminobenzylamines and 2-aminobenzoketones; (ii) using *N*-arylamidines as a substrate, quinazoline derivatives have been developed, in which pre-activation at the ortho-position of the N-aryl ring is not necessary (Figure 2).

**Figure 3:**
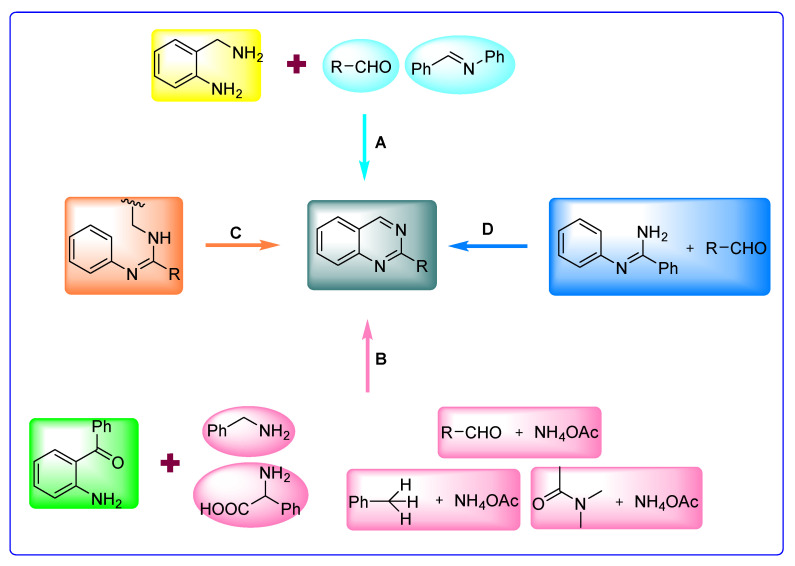
Classical strategies for the synthesis of quinazolines.

These methods mainly involve: (A) condensation of 2-aminobenzylamines with aldehydes followed by oxidation with oxidants such as MnO_2_, DDQ, and sodium hypochlorite [41,42,43]; a reaction between 2-aminobenzylamines with imines in the presence of K_2_S_2_O_8_ as oxidant [44], (B) oxidative cyclization of 2-aminobenzophenones with benzylamines [45]; two-component oxidative decarboxylative amination reaction of 2-aminobenzophenones with α-amino acid catalyzed by iodine/TBHP [46]; one-pot three-component reaction of 2-aminobenzophenones, aldehyde, and NH_4_OAc in mixtures of maltose-dimethylurea (DMU)-NH_4_Cl [47]; KI-catalyzed three-component reaction of 2-aminobenzophenones, methylarenes, and NH_4_OAc [48]; NS/TBHP-system-catalyzed reaction with NH_4_OAc as a nitrogen source and dimethylacetamide (DMA) as a one carbon synthon [49], (C) intramolecular oxidative cyclization of N-arylated amidines [50], and (D) microwave-promoted synthesis of quinazolines from N-arylamidines and aldehydes [51] (Figure 3). Despite the efficacy of these protocols, they also suffer from inevitable disadvantages such as the use of excessive quantities of dangerous peroxide reagents, toxic solvents, toxic oxidants such as NaOCl, longer reaction times, elevated temperatures, and requiring strong and hazardous oxidizing agents. Consequently, rapid growth has been witnessed in the last few years regarding the metal-free synthesis of quinazolines, and the methods are compiled in this review. Several reviews have been presented on the synthesis and biological activity of quinazolines [52,53]. This review primarily focuses on the recent progress achieved in the transition-metal-free synthesis of quinazolines and covers the literature from 2015 to 2022. In addition, highlighting the advances made so far, we also point out the limitations associated with them. Furthermore, the article is organized based on the substrates used in a chronological order.

## 2. Synthesis of Quinazolines

### 2.1. From O-Functionalized Anilines/Amines

**Scheme 1:**
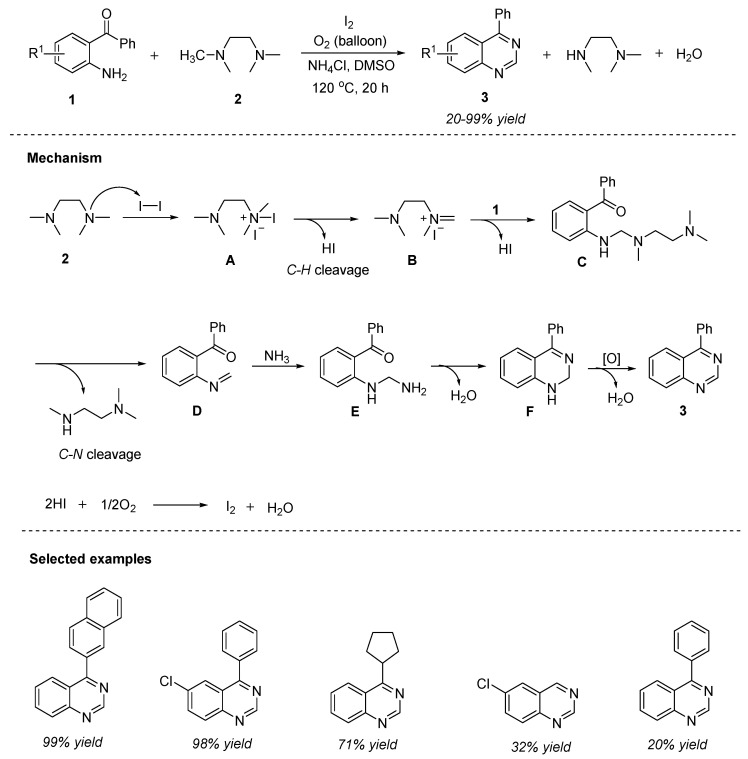
Iodine-catalyzed aerobic oxidative synthesis of quinazolines.

In the recent years, transition-metal-free intra- or intermolecular oxidative C(sp^3^)-H amination has upsurged as a significant approach for the direct C-N bond formation [54]. Moreover, cost-effectiveness, step economy, and less toxicity are the benefits associated with this strategy. Liu et al. (2015) reported a rarely explored carbon source viz., tertiary amines for the C-N bond formation in quinazolines via an iodine-catalyzed C(sp^3^)-H amination [55]. The tandem reaction of 2-aminobenzophenone **1** and *N*-methylamines **2** (carbon source) in the presence of iodine as a catalyst, ammonium chloride as the nitrogen source, utilizing molecular oxygen as an oxidant in DMSO at 120 °C for 12 h furnished quinazolines **3** in 20–98% yields (Scheme 1). Under the optimized conditions, aromatic amines displayed greater reactivity than aliphatic amines. The major advantages of this approach involve: peroxide-free, broad substrate scope, operationally simple, oxygen as the green oxidant, metal-free, and easy scalability up to 10 mmol with 90% yield.

The plausible reaction mechanism depicted in Scheme 1 suggested that the initial reaction of tetramethylethylenediamine **2** (TMEDA) with iodine generated an aminium iodide **A**, which then gives an iminium iodide **B** by the removal of molecular hydrogen iodide. Nucleophilic addition of **1** to **B** forms an intermediate **C** by the simultaneous removal of another HI. Later, intermediate **E** was formed by the nucleophilic addition of ammonia to **D**. In the last step, **E** undergoes a tandem condensation–oxidation to provide the desired product **3**. Simultaneously, the I_2_/I catalytic cycle is completed in the form of iodine regeneration via the oxidation of in situ generated HI by oxygen.

**Scheme 2:**
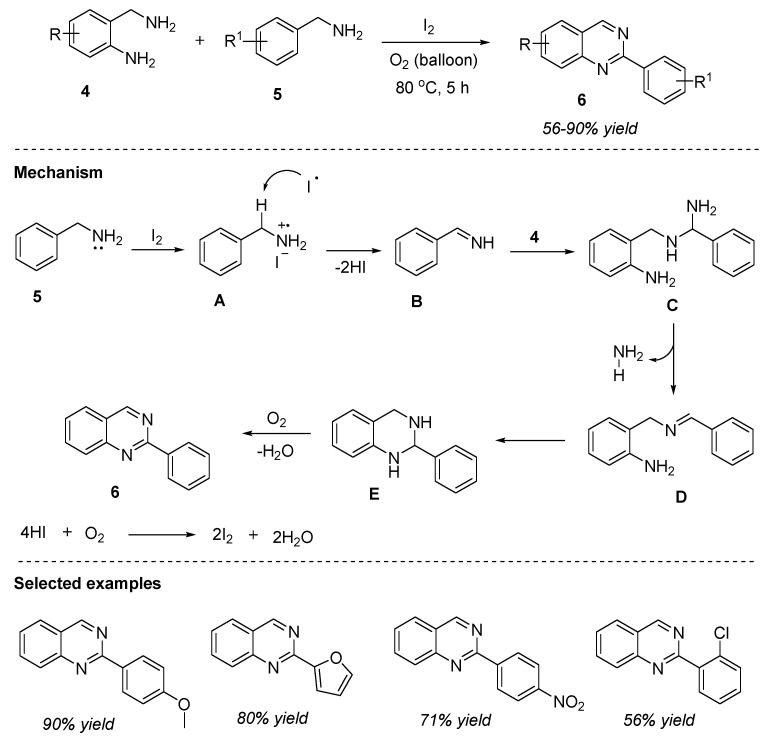
Iodine-catalyzed oxidation for the synthesis of quinazolines.

Likewise, Bhanage et al. (2016) disclosed a green and sustainable method for the construction of 2-arylquinazolines **6** from 2-aminobenzylamine **4** and *N*-mono substituted benzylamines **5** in the presence of molecular iodine at 80 °C for 5 h under O_2_ balloon (Scheme 2) [56]. The optimized reaction conditions worked well for a variety of substrates including electron-donating and electron-withdrawing groups and provided quinazolines **6** in 56–90% yields. Additive-free, solvent-free, transition-metal-free, utilizing oxygen as the green oxidant, shorter reaction times, and simple operation conditions are the major benefits of this approach.

An insight into the reaction mechanism as shown in Scheme 2 suggested that the generation of the radical intermediate initiated the reaction, followed by the formation of imine **B**. Later, imine **B** reacted with 2-aminobenzylamine **4** to form intermediates **C**, **D**, and **E** successively. In the last step, **E** undergoes dehydrogenative aromatization to afford the desired product **6**. Simultaneously, iodine is regenerated by the reaction of HI with oxygen.

**Scheme 3:**
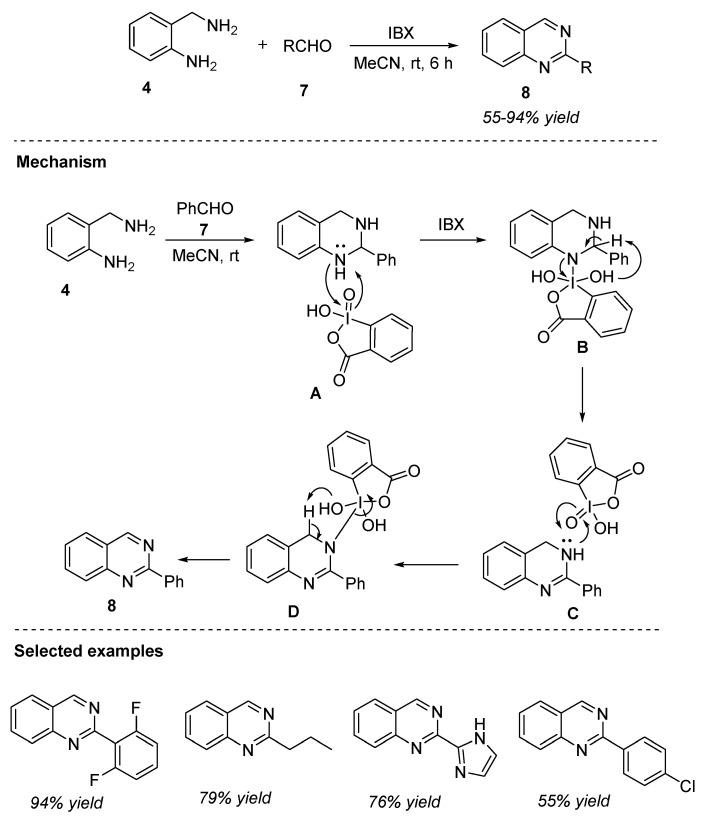
IBX-mediated reaction for the synthesis of quinazolines.

In the same year, Sen et al. (2016) developed the facile synthesis of quinazolines through the o-iodoxybenzoicacid (IBX)-mediated reaction of 2-aminobenzylamine **4** and aldehydes **7** in acetonitrile at room temperature for 6 h (Scheme 3) [57]. Various aryl, heteroaryl, and alkyl aldehydes **7** could be transformed to the corresponding 2-substitued quinazolines **8** in 55–94% yields.

The plausible mechanism proposed by the authors is presented in Scheme 3. The initial attack of nitrogen in intermediate A on the iodine center of IBX, followed by reduction generated the dihydroquinazoline **C**. Similarly, attack by the nitrogen in dihydroquinazoline **C** on IBX afforded quinazoline **8** via an intermediate **D**.

**Scheme 4:**
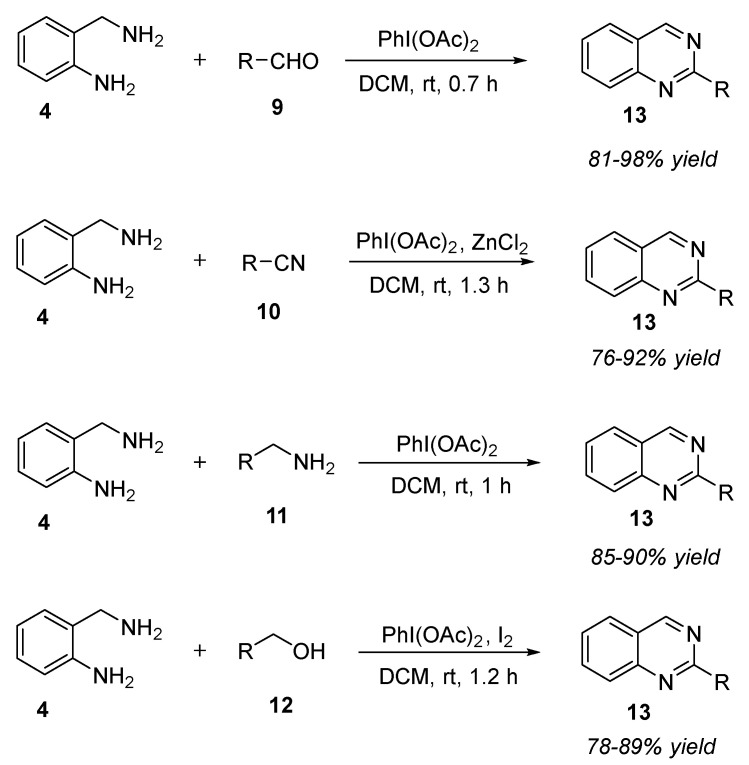
PIDA-mediated reaction for the synthesis of quinazolines.

Hypervalent iodine reagents provide a metal-free reaction condition which is essential in the field of medicinal chemistry for the synthesis of pharmacologically important heterocycles. One such example is iodobenzene diacetate (PIDA). Less toxic effect and easy availability made the oxidant be used widely in chemical transformations [58]. Das et al. (2017) disclosed an interesting approach for the synthesis of 2-substitued quinazolines **13** by the reaction of 2-aminobenzylamine **4** with a variety of aldehydes **9**, nitriles **10**, aliphatic/aryl amines **11**, and aliphatic/aryl alcohols **12** in the presence of PIDA as the key reagent, dichloromethane as the solvent, at room temperature for 0.7–1.3 h (Scheme 4) [59]. In the case of alcohols **12** and nitriles **10**, catalytic amounts of molecular iodine and zinc chloride, respectively, were employed to facilitate the reaction. A diverse range of aliphatic, aryl, or heteroaryl groups afforded 2-substitued quinazolines **15** in 76–98% yields. The major advantages of this strategy included: (a) readily available starting materials, (b) broad substrate scope, (c) employing a common oxidant for different substrates, (d) mild reaction conditions, (e) absence or presence of additives, and (f) product-selective reaction with no side products.

**Scheme 5:**
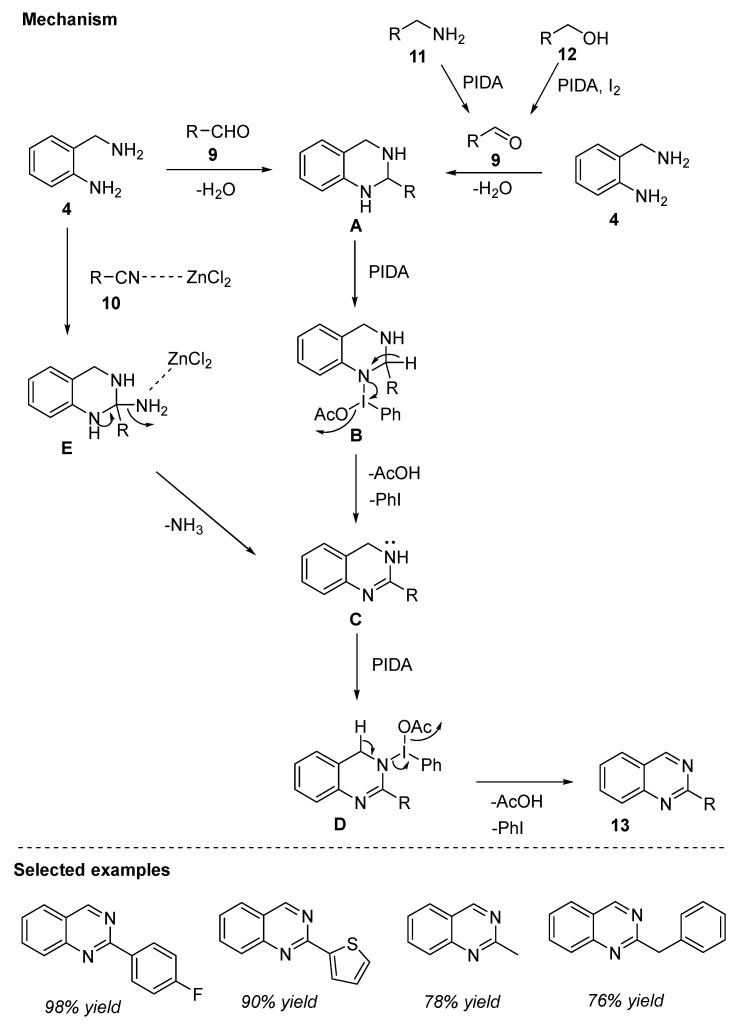
PIDA-mediated reaction mechanism for the synthesis of quinazolines.

The proposed mechanism as depicted in Scheme 5 suggested that the reaction between 2-aminobenzylamine **4** and aldehyde **9** resulted in the generation of intermediate **A**. **A** upon reaction with PIDA led to the formation of intermediate **B**. Elimination followed by the attack with another molecule of PIDA afforded the desired product via intermediates **C** and **D**. Oxidation of arylamine **11** and arylalcohol **12** derivatives in the presence of PIDA and PIDA/I_2_, respectively, generated the corresponding aldehydes **9**. Aldehydes **9** then underwent a similar course of mechanism to furnish quinazoline **13**. In the case of nitriles **10**, coordination of ZnCl_2_ with the cyano group [60] provided the intermediate **E**, which then liberates ammonia in the presence of ZnCl_2_ to form intermediate **C**. Quinazoline **13** is formed in the subsequent steps by following a similar mechanism as detailed earlier.

**Scheme 6:**
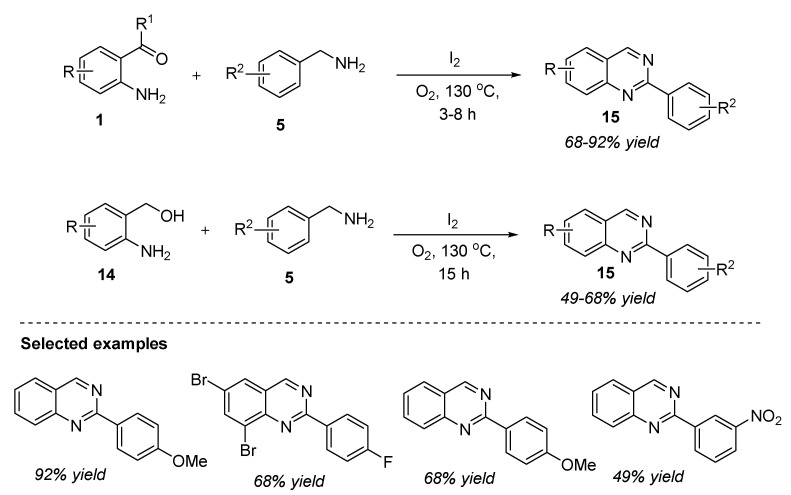
Molecular-iodine-catalyzed synthesis of quinazolines.

Molecular iodine, a mild Lewis acidic reagent, is known to be an established catalyst in organic reactions. It performs several synthetic transformations such as Michael addition, iodination, oxidation, Prins-related reactions, protection–deprotection of functional groups, and functionalization of alcohols [61]. In spite of the recently reported transition-metal-free protocols for the functionalization of benzylic sp^3^ C-H bonds, there remains a need to develop new strategies for the synthesis of 2-arylquinazolines by overcoming the limitations of previous reports. In this connection, Bhanage et al. (2018) documented a green and sustainable approach for the synthesis of quinazolines via benzylic sp^3^ C-H bond amination [62]. The reaction of 2-aminobenzophenones **1** with aryl amines **5** catalyzed by molecular iodine under O_2_ atmosphere at 130 °C for 3–8 h afforded 2-arylquinazolines **15** in 68–92% yields (Scheme 6). Furthermore, the reaction of 2-aminobenzyl alcohol **14** and benzylamine **5** in the presence of molecular iodine under O_2_ at 130 °C for 15 h delivered 2-arylquinazolines **15** in 49–68% yields (Scheme 6). Broad substrate scope, use of oxygen as the green oxidant, additive- and solvent-free conditions, and lack of aqueous workup are the major benefits of this protocol. Aliphatic and unsaturated amines failed to form the desired product, and this remains the only limitation associated with this strategy.

**Scheme 7:**
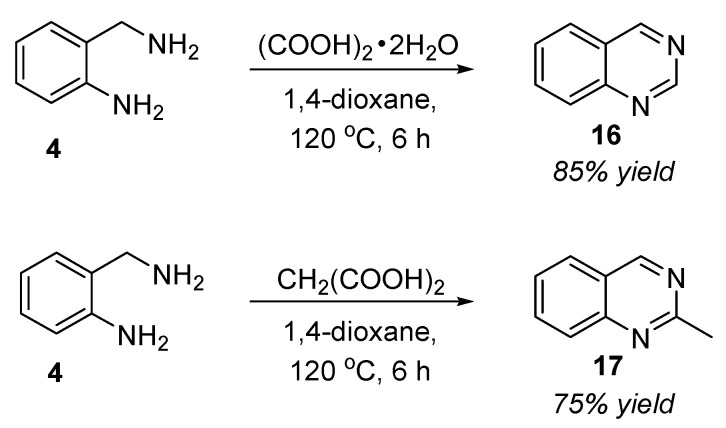
Oxalic-acid-dihydrate/Malonic-acid-mediated reaction for the synthesis of quinazolines.

In the same year, Das et al. (2018) developed an oxidant-, base-, and metal-free approach for the synthesis of quinazolines by the reaction of 2-aminobenzylamine **4** with oxalic acid dihydrate or malonic acid in 1,4-dioxane at 120 °C for 6 h via an in situ condensation and oxidation process [63]. Quinazoline **16** and 2-methylquinazoline **17** were obtained in 75–85% yields (Scheme 7). Utilizing oxalic acid dihydrate as the in situ C1 source, easy handling, atom economy, and greener reaction conditions are the merits of this protocol.

**Scheme 8:**
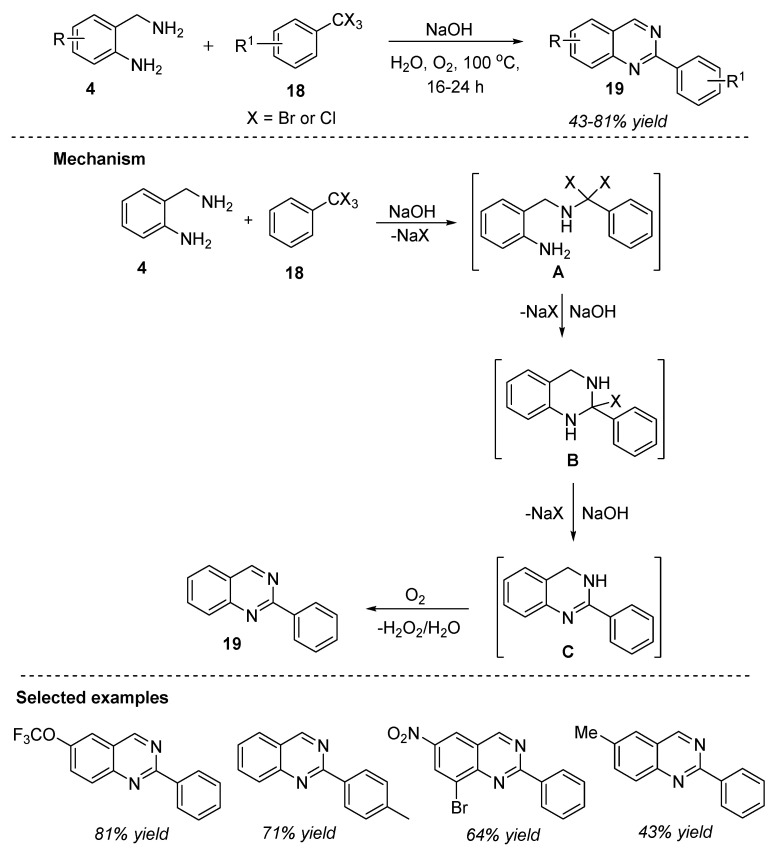
Base-promoted reaction for the synthesis of quinazolines.

In contrast to the above-mentioned strategy, Cho et al. (2018) reported a sustainable methodology for the synthesis of 2-arylquinazolines **19** from 2-aminobenzylamines **4** and α,α,α-trihalotoluenes **18** in the presence of sodium hydroxide and molecular oxygen in water at 100 °C for 16–24 h (Scheme 8) [64]. A variety of electron-donating and electron-withdrawing groups on the substrates delivered quinazolines **19** in 43–81% yields under optimized reaction conditions. The important uses of this synthesis method include: readily available substrates, green and renewable solvent, isolation of products by simple crystallization, formation of benign by-product (NaCl or NaBr), chemical oxidant or additive-free conditions, and avoiding huge solvent-consuming work-up procedures.

The reaction mechanism outlined in Scheme 8 suggest that the initial base-promoted intermolecular substitution of α,α,α-trihalotoluene **18** by 2-aminobenzylamine **4** led to the generation of intermediate **A**. Later, base-mediated intramolecular substitution followed by elimination formed the intermediate **C**, which upon oxidation afforded the desired product, quinazoline **19**.

**Scheme 9:**
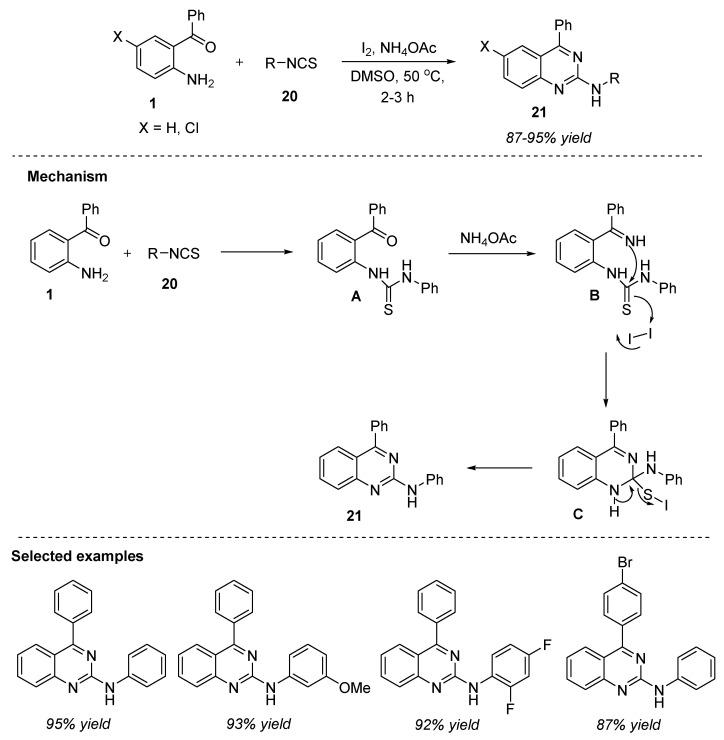
I_2_-catalyzed oxidative synthesis of quinazolines.

Palakodety et al. (2019) reported the metal-free protocol for the construction of *N*,4-disubstituted quinazolin-2-amine **21** from 2-aminobenzophenone **1**, phenylisothiocyanate **33**, and ammonium acetate as the nitrogen source in the presence of iodine in dimethylsulfoxide at 50 °C for 2–3 h (Scheme 9) [65]. Under the optimized conditions, a wide range of electron-donating and electron-withdrawing groups present on isothiocyanate **20** and 2-aminobenzophenone **1** offered the corresponding *N*,4-disubstitued quinazolin-2-amines **21** in 87–95% yields. Moreover, *meta* and *ortho*-substituted 2-aminoaryl ketones **1** did not tolerate the established conditions due to the steric effect and this remains a drawback to this strategy. Readily available inexpensive substrates, operational simplicity, shorter reaction times, and excellent yields are the key benefits of this methodology.

The plausible mechanism is shown in Scheme 9. Initially, 2-aminobenzophenone **1** undergoes condensation with phenylisothiocyanate **20** to afford the thiourea intermediate **A**. Then, **A** reacts with ammonium acetate to form intermediate **B**, which upon intramolecular attack of the imine NH group onto the thiocarbonyl carbon with concomitant trapping of S anion resulted in the generation of cyclized product **C**. Aromatization of **C** delivered the desired product **21** with subsequent removal of S and hydrogen iodide.

**Scheme 10:**
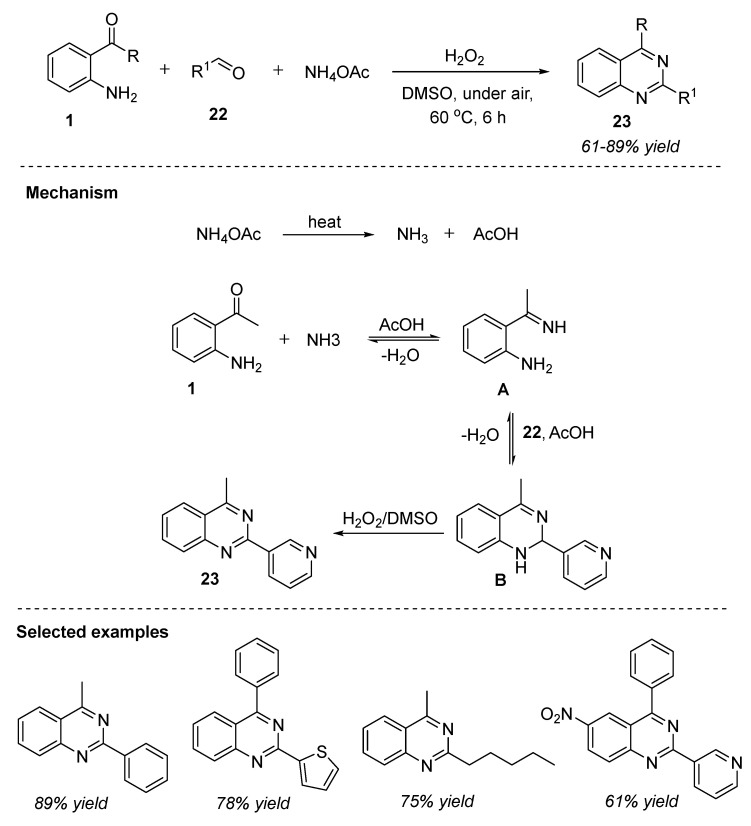
H_2_O_2_-mediated reaction for the synthesis of quinazolines.

Hydrogen peroxide has emerged as an ideal oxidant for liquid-phase green chemistry owing to its non-toxic, atom efficient, and environmentally benign properties [66]. Furthermore, hydrogen peroxide oxidant usually does not require temperatures over 100 °C to carry out oxidation transformations. Phan et al. (2020) presented hydrogen-peroxide-mediated one-pot three-component reaction of 2-aminoaryl ketones **1**, aldehydes **22**, and ammonium acetate as the nitrogen source for the efficient synthesis of 2,4-substituted quinazolines **23**. The transformation proceeded readily in the presence of hydrogen peroxide as an oxidant and DMSO as the solvent under air at 60 °C for 6 h (Scheme 10) [67]. The highlighted benefits of this approach are: readily available and atom-efficient H_2_O_2_, mild reaction conditions, transition-metal-free conditions, and broad substrate scope. Under the optimized conditions, various 2-aminobenzophenones **1** coupled with aromatic, heteroaromatic, and aliphatic aldehydes **22** and delivered the corresponding products **23** in 61–89% yields. This is the first report utilizing hydrogen peroxide as the green oxidant for the synthesis of 2,4-substituted quinazolines **23**.

The proposed pathway for the transformation was shown in Scheme 10. In the first step, ammonium acetate was decomposed to ammonia and acetic acid upon heating. Later, condensation between 2-aminoacetophenone **1** with NH_3_ generated an imine intermediate **A**. Subsequent condensation between the amino group of **A** with aldehyde **22** followed by nucleophilic cyclization provided the dihydroquinazoline intermediate **B**. In the presence of H_2_O_2_ and DMSO, intermediate **B** was rapidly oxidized to furnish the final product, quinazoline **23**.

**Scheme 11:**
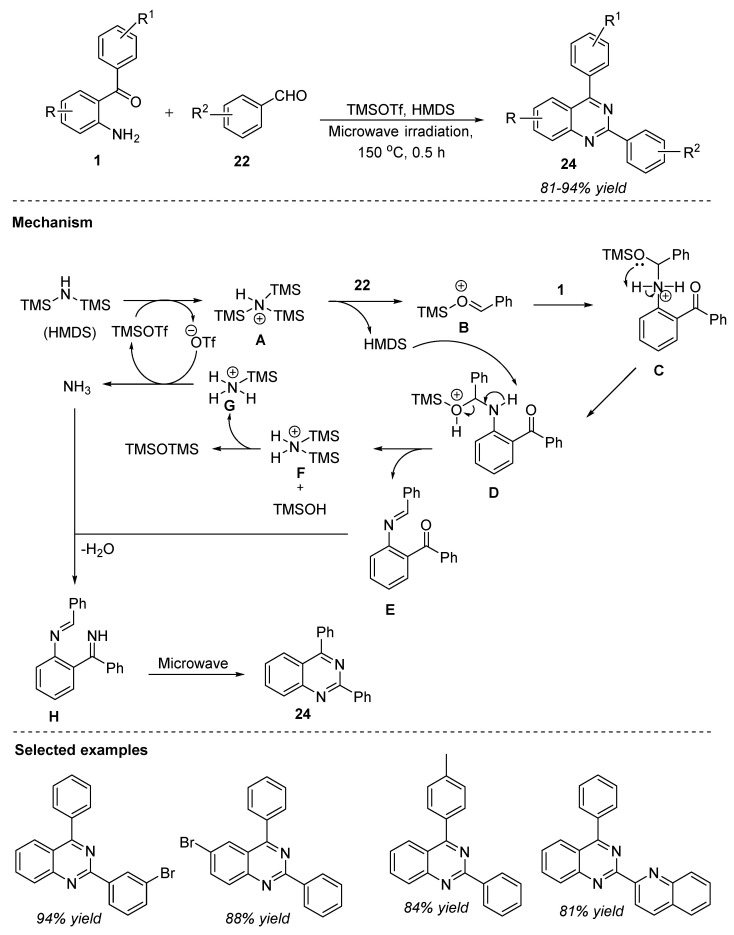
TMSOTf-HMDS-catalyzed synthesis of quinazolines.

Hexamethyldisilazane (HMDS) is a commercially available stable reagent employed for the silylation reactions to increase the silylating power by utilizing several catalysts [68]. Wang et al. (2020) developed an efficient and mild synthetic route for the facile synthesis of substituted quinazolines. The reaction between 2-aminobenzophenones **1** and benzaldehydes **22** in the presence of catalytic trimethylsilyl trifluoromethanesulfonate (TMSOTf) and HMDS in which gaseous ammonia was formed in situ under neat, and microwave irradiation at 150 °C for 0.5 h, delivered substituted quinazolines **24** in 81–94% yields (Scheme 11) [69]. An array of aromatic benzaldehydes **22** substituted with electron-donating and electron-withdrawing groups and heterocyclic carbaldehydes were well tolerated to afford the corresponding quinazolines **24**. Moreover, the structures of quinazolines **24** were also confirmed by single-crystal X-ray crystallography. Absence of unnecessary waste due to conventional reaction conditions viz., microwave irradiation, shorter reaction times, gram-scale synthesis with 90–91% yields, broad substrate scope, and good to excellent yields are the merits of this protocol.

The plausible mechanism, as shown in Scheme 11, indicated that in the initial step, the TMSOTf and HMDS system could form a complex **A** with the liberation of the triflate anion. The complex **A** reacts with benzaldehyde **22** to generate an oxonium intermediate **B**. Intermediate **C** was then formed by the intermolecular addition of 2-aminobenzophenone **1** with **B**. Deprotonation of intermediate **D** mediated by HMDS resulted in intermediate **E**. Simultaneous reaction between complex **F** and TMSOH led to the silylated product TMSOTMS ether and complex **G**, which upon further transformation, liberated gaseous ammonia by triflate anion. At the same time, TMSOTf was regenerated. Later, intermediate **E** underwent imination followed by oxidation with in situ ammonia, producing intermediate **H**. Finally, microwave irradiation of **H** furnished the desired product quinazoline **24**.

**Scheme 12:**
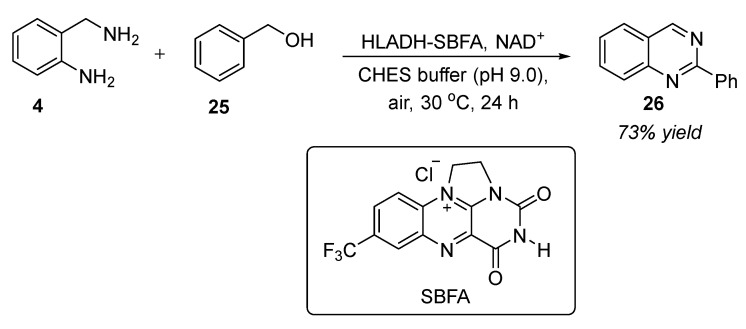
HLADH-SBFA-catalyzed synthesis of quinazolines.

Among the biological cascade systems, chemoenzymatic synthesis methods have drawn considerable attention by the integration of biocatalysis with the versatile reactivity of chemocatalysis [70]. Noteworthy advantages of the chemoenzymatic cascade process include: cost-effectiveness, simple operation conditions, improved selectivity, omitting isolation and purification of intermediates, lower waste generation, and higher reaction yields. In this connection, Zhu et al. (2022) reported the first example of the chemoenzymatic synthesis of quinazolines [71]. The condensation reaction of 2-aminobenzylamine **4** and benzyl alcohol **25** in the presence of the HLADH-SBFA catalytic system and maintaining the pH 9.0 by CHES buffer, under air at 30 °C for 24 h, furnished 2-phenylquinazoline **26** in 73% yields (Scheme 12). In this reaction, synthetic bridged flavin analog (SBFA) performed regeneration of both NAD(P)^+^ and the cofactor during the alcohol dehydrogenase (ADH)-catalyzed process of alcohol to aldehyde. HLADH was used as a biocatalyst and SBFA served as a bifunctional chemocatalyst. Mild reaction conditions, employing bifunctional organocatalyst SBFA, and utilizing O_2_ as an external oxidant, serve as a green and sustainable alternatives in this strategy.

**Scheme 13:**
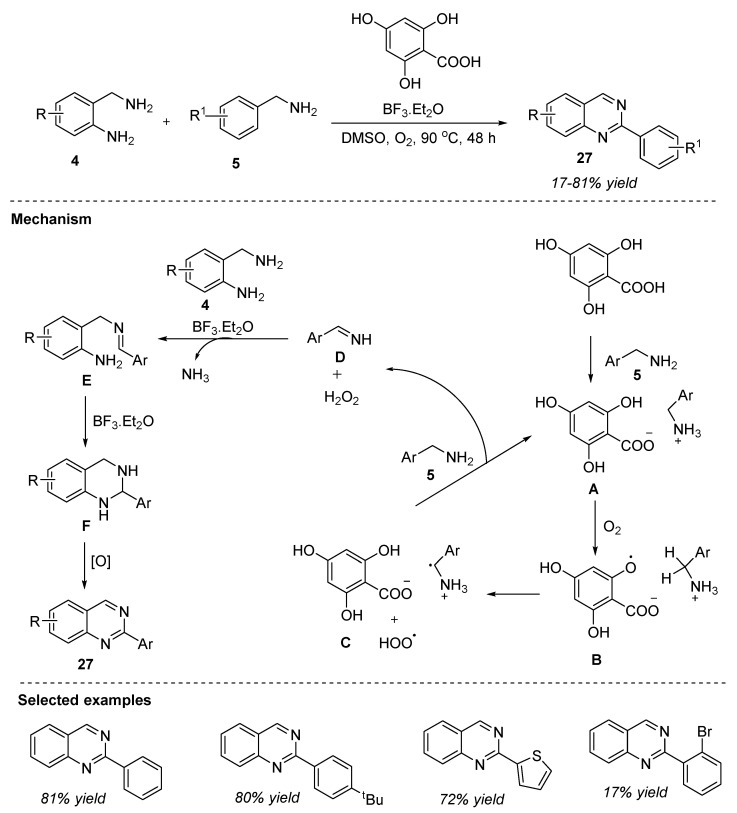
4,6-Dihydoxysalicylic-acid-catalyzed synthesis of quinazolines.

Another interesting strategy was put forth by Ogawa et al. (2022) for the synthesis of 2-substituted quinazolines by the 4,6-dihydoxysalicylic-acid-catalyzed oxidative condensation. The organocatalytic oxidation of 2-aminobenzylamines **4** and benzylamines **5** employing 4,6-dihydoxysalicylic acid as the catalyst and a catalytic amount of Lewis acid, BF_3_.Et_2_O in DMSO under O_2_ atmosphere at 90 °C for 48 h, afforded quinazoline derivatives **27** in 17–81% yields with an excellent environmental (E)-factor (Scheme 13) [72]. Broad substrate scope, gram-scale synthesis, clean reaction system, and easy isolation are the noteworthy uses of this approach.

The organocatalytic oxidative reaction mechanism was depicted in Scheme 13. In the first step, benzylamine **5** reacted with 4,6-dihydrosalicyclic acid to form salt **A** which generated aryloxy radical **B** and HOO• via hydrogen abstraction by O_2_. In the next step, intramolecular hydrogen abstraction of the benzyl group led to the formation of radical cation **C** [73], which then gave imine **D** under the action of HOO•. Imine **D** then underwent an amino group exchange reaction with 2-aminobenzylamine **4** in the presence of BF_3_.Et_2_O to generate intermediate **E**. Later, **F** was formed by the intramolecular cyclization of **E**, enhanced by BF_3_.Et_2_O. In the last step, oxidative aromatization of **F** delivered the desired product, 2-arylquinazoline **27**.

**Scheme 14:**
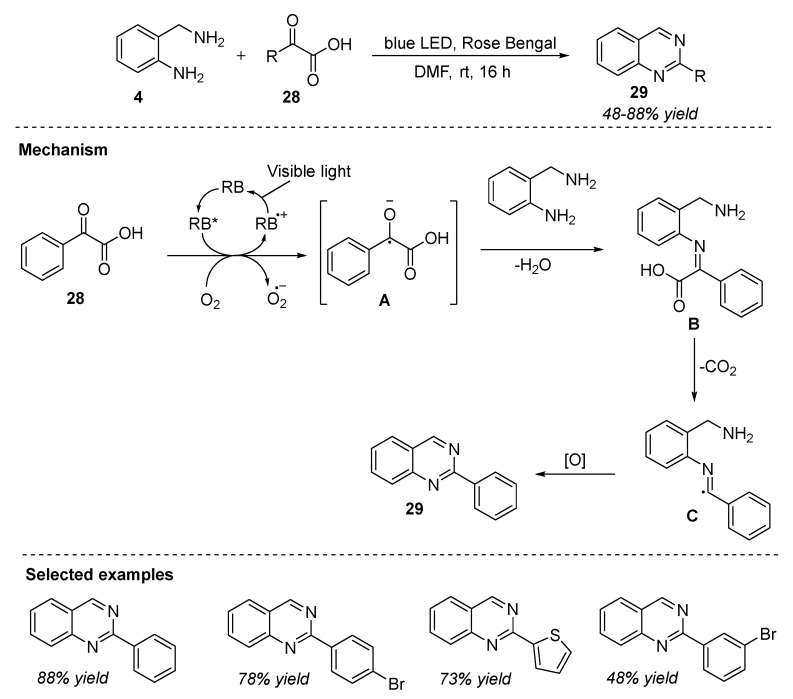
Visible-light-photocatalyzed synthesis of quinazolines.

Visible-light photocatalysis has received considerable attention owing to its ability to carry out redox catalysis via a single electron transfer process under mild conditions. Le et al. (2022) synthesized quinazolines by the condensation of 2-aminobenzylamines **4** and α-ketoacids **28** followed by blue-light LED irradiation at room temperature. The reaction of 2-aminobenzylamines **4** and α-ketoacids **28** in the presence of an organic dye photocatalyst, Rose Bengal, in DMF under air in blue-light LED irradiation at room temperature for 16 h gave the corresponding quinazolines **29** in 26–88% yields (Scheme 14) [74]. Transition-metal-free, additive-free, external-oxidizing-agent-free, and simple and mild reaction conditions made this a green and environmentally friendly process. A variety of electron-donating, electron-withdrawing, electrically neutral, and heterocyclic substituents on α-ketoacids worked well under the optimized conditions.

The reaction mechanism proposed by the authors was shown in Scheme 14. Initially, benzoylformic acid **28** reacted with excited Rose Bengal under blue-light irradiation to generate anion intermediate **A**, which then dehydrates with 2-aminobenzylamine **4** to give another intermediate **B**. Then, **B** undergoes decarboxylation to yield free radical intermediate **C**, followed by oxidation and cyclization which furnished quinazoline **29**.

### 2.2. From Amidines and Azirines

**Scheme 15:**
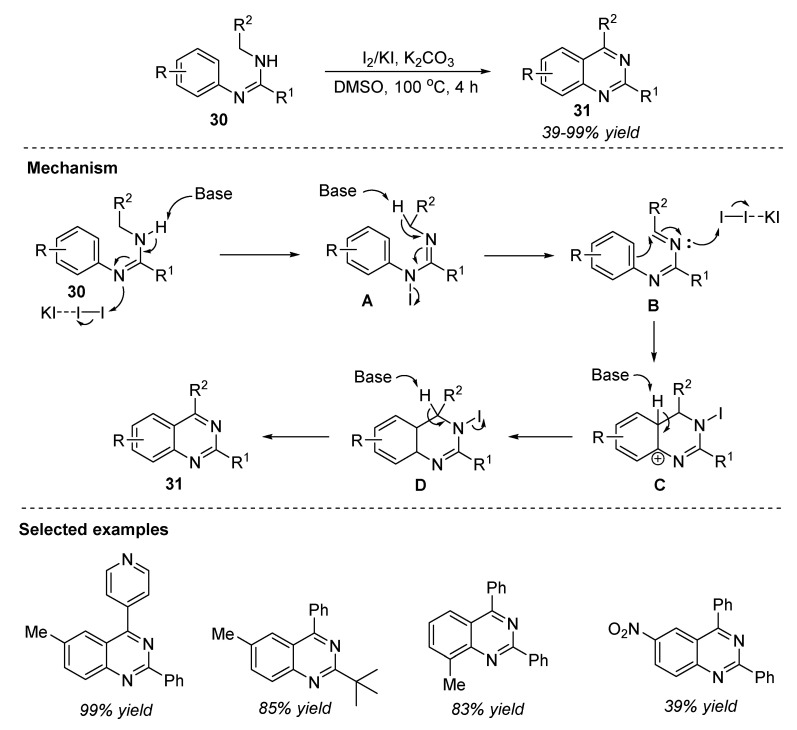
I_2_/KI-promoted oxidative synthesis of quinazolines.

Molecular iodine is a less toxic and inexpensive oxidant widely used for the oxidative C-X (X = C, N, O, or S) bond construction from C-H bonds for the synthesis of heterocyclic compounds. In this relation, Chang et al. (2016) reported an I_2_/KI-promoted oxidative C-C bond formation reaction from C(sp^3^)-H and C(sp^2^)-H bonds from *N*,*N*’-disubstituted amidines **30** in the presence of potassium carbonate in DMSO at 100 °C for 4 h to provide quinazolines **31** in 39–99% yields (Scheme 15) [75]. The reaction proceeded smoothly with both electron-donating and electron-withdrawing groups on the *N*’-phenyl rings and gave the corresponding products **31**. Gram-scale synthesis with a 61% yield, readily prepared starting precursors, and broad substrate scope are the merits of this protocol.

The mechanism of oxidative cyclization is depicted in Scheme 15. Initially, the oxidation of *N*-aryl amidine **30** by molecular iodine in the presence of base K_2_CO_3_ led to an imine intermediate **B**. Next, iodine-mediated iodocyclization of **B** generated a *N*-iodo species **C** with a new C–C bond. Base-promoted deprotonation followed by elimination of one molecule of HI, delivered the quinazoline **31**.

**Scheme 16:**
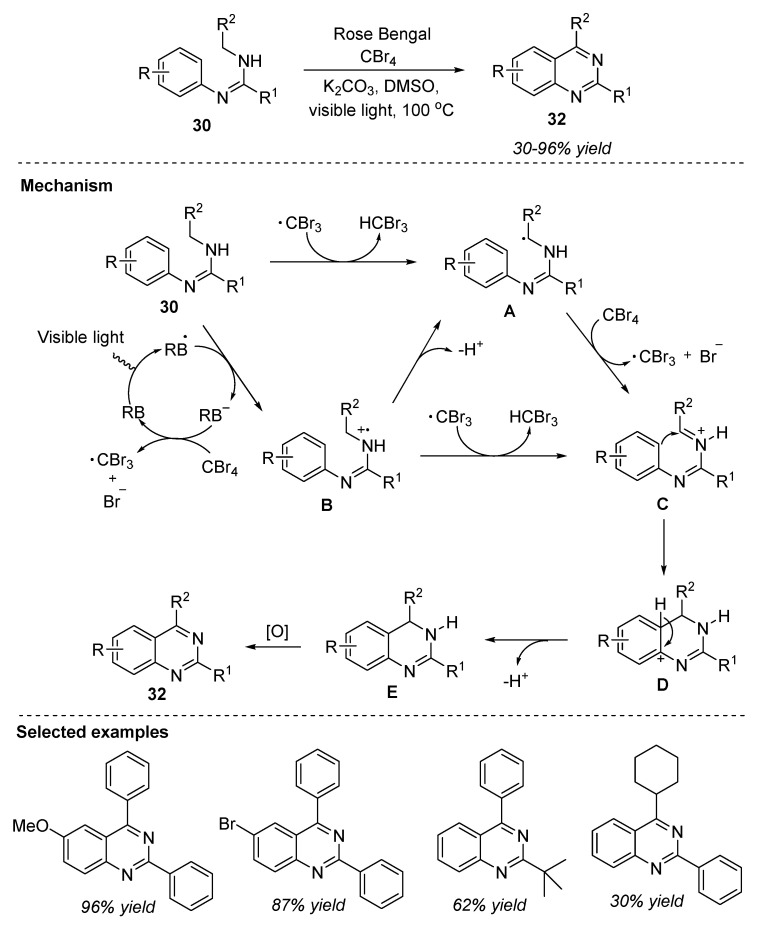
Visible-light-mediated oxidative synthesis of quinazolines.

In the same year, Tang et al. (2016) disclosed a fast catalytic synthesis of multi-substituted quinazolines from amidines via visible-light-mediated oxidative C(sp^3^)-C(sp^2^) bond formation. Metal-free oxidative coupling of amidine **30** in the presence of Rose Bengal as a photoredox organocatalyst, CBr_4_ as an oxidant, and K_2_CO_3_ as the base, in DMSO as the solvent at 100 °C under 18 W fluorescent lamp as the visible-light source provided quinazolines **32** in 30–96% yields (Scheme 16) [76]. The major advantages of this reaction include: lower catalyst loading, improved efficacy of reaction, and tolerating a wide range of functional groups in substrates.

The plausible reaction mechanism was demonstrated in more than one pathway (Scheme 16) [77]. In the absence of Rose Bengal, CBr_4_ was cracked into CBr_3_ radical and Br radical under visible-light irradiation [78]. CBr_3_ radical abstracted a hydrogen atom from another molecule of benzimidamide to yield α-amino radical intermediate **A**, which then entered the radical chain process with CBr_4_ to give iminium ion **C**. Intramolecular Friedel-Craft reaction of **C** generated an intermediate **D**, which upon dehydrogenation followed by aromatization delivered the desired product **32**. In another pathway, quenching of visible-light-excited Rose Bengal (RB*) by benzimidamide led to the formation of Rose Bengal radical anion (RB•−) and radical cation **B**. The photoredox cycle was completed by the formation of a superoxide radical anion by the transfer of an electron to CBr_4_ and regeneration of Rose Bengal. Furthermore, the radical cation **B** gave up a hydrogen atom to the radical anion to generate CBr_3_ radical, bromide anion, and radical intermediate **A**. The CBr_3_ radical abstracted a hydrogen atom from radical cation **B** and formed iminium ion **C**, in another pathway.

**Scheme 17:**
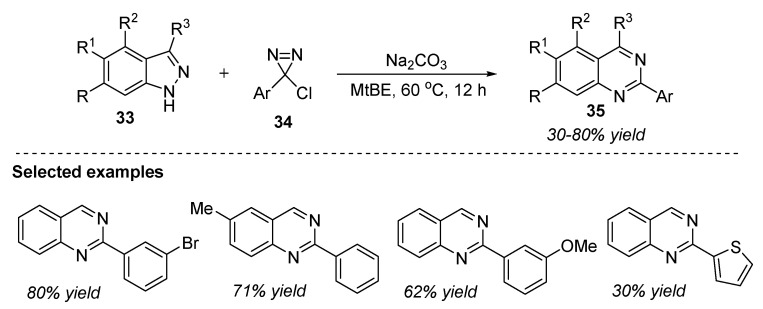
Chlorodiazirine-mediated reaction for the synthesis of quinazolines.

An interesting approach was developed by Levin et al. (2022) for the construction of quinazoline scaffolds by the selective cleavage of the N-N bond of the indazole core **33** [79]. This chlorodiazirine-**34**-mediated reaction provides unified access to quinazolines **35** in the presence of sodium carbonate and methyl tert-butyl ether (MtBE) at 60 °C for 12 h (Scheme 17). Indazoles **33** were tolerant towards a wide range of alkyl substitutions. Moreover, free alcohol, thiophene, and halogen groups substituted in various positions also gave quinazolines **35** in 30–80% yields. The two major limitations associated with this strategy include: (a) substrates with less solubility in refluxing MtBE displayed poor reactivity, and (b) nucleophilicity of diazole was deactivated due to inductive withdrawal and precludes productive reactivity.

### 2.3. From Nitriles

**Scheme 18:**
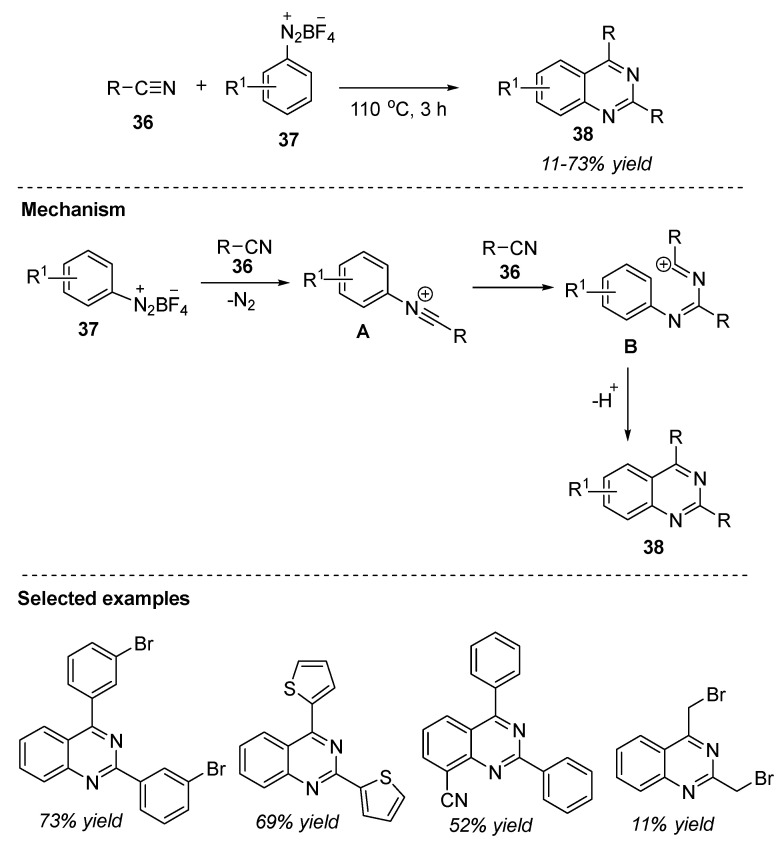
(2 + 2 + 2) Annulation reaction for the synthesis of quinazolines.

Liu et al. (2017) reported a (2+2+2) modular synthesis of multi-substituted quinazolines by the direct reaction of two equivalents of nitriles **36** with aryldiazonium salts **37**. The reaction of nitriles **36** and aryldiazonium tetrafluoroborate **37** at 110 °C for 3 h provided quinazolines **38** in 11–73% yields (Scheme 18) [80]. Under the optimized conditions, several aryldiazonium tetrafluoroborates **37** with electron-donating and electron-withdrawing groups at different positions in the ring were reacted smoothly to afford the corresponding products **38**. Similarly, *ortho*- or *para*-substituted phenyldiazonium salts **37** gave quinazolines in good yields with the exception of nitro substituent. Moreover, aliphatic, aromatic, and heteroaromatic nitriles **36** served as suitable substrates for this annulation. Readily available substrates, shorter reaction times, one-pot, neat and metal-free conditions, gram-scale synthesis with 74% yields, flexibility in substitution patterns, atom-efficient, and easy handling procedures are the benefits of this strategy.

The authors proposed that the *N*-arylnitrilium ion **A** was generated in situ by the reaction of aryldiazonium salt **37** and nitrile **36**. The *N*-arylnitrilium ion **A** undergoes a further reaction with another nitrile moiety **36** via tandem addition/electrophilic cyclization to furnish 2,4-disubstituted quinazolines **38** in an atom-economic way (Scheme 18).

**Scheme 19:**
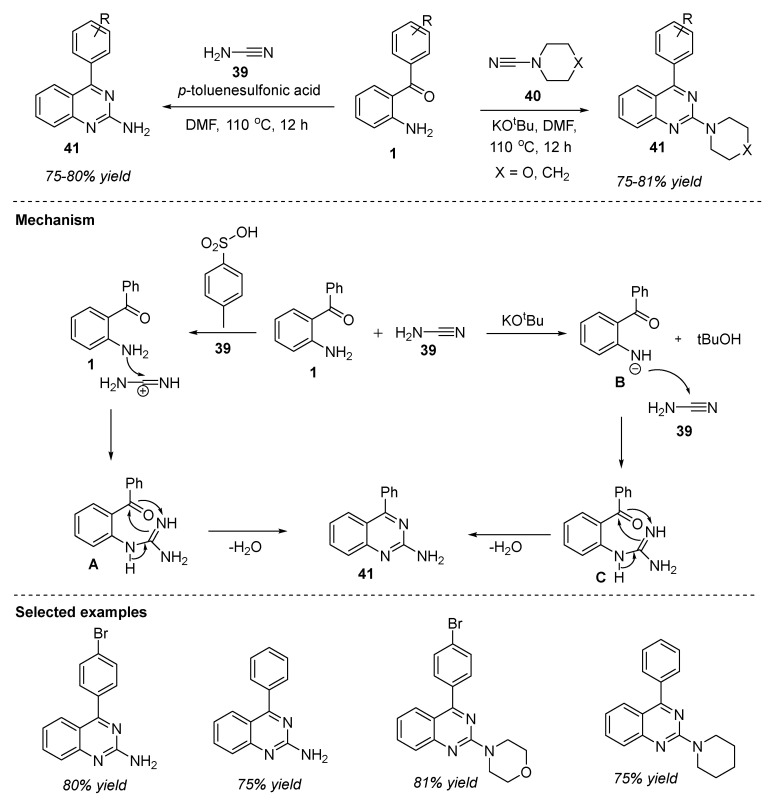
PTSA/KO^t^Bu-mediated reaction for the synthesis of quinazolines.

North et al. (2017) reported a metal-free oxidative annulation reaction for the synthesis of 2-aminated quinazolines **41** by the reaction of 2-aminobenzophenone **1** with unsubstituted cyanamide/4-morpholinecarbonitrile **39/40** in the presence of *p*-toluene sulfonic acid (PTSA)/potassium tertiary butoxide (KO^t^Bu) in DMF at 110 °C for 12 h (Scheme 19) [81]. The reaction proceeded with a variety of strong electron-withdrawing and unsubstituted aromatic rings to deliver quinazolines **41** in 75–81% yields. The plausible reaction mechanism for the construction of 2-aminoquinazolines **41** mediated by an acid or base is shown in Scheme 19. The initial step is the protonation of nitrogen in the cyano group of **39** by the weak acid PTSA resulting in the increased electrophilic nature of the cyanamide carbon [82]. In the next step, amine **1** attacks the electrophilic carbon generating the intermediate, amidine **A**. Simultaneous cyclization followed by elimination of the water molecule from intermediate **A** delivered the product 2-aminoquinazoline **41**. On the other hand, base-mediated proton removal of amine **1** led to the formation of a strong nucleophile **B** which then reacts with the cyanamide carbon **39** followed by ring closure, resulting in the formation of the desired product **41**.

**Scheme 20:**
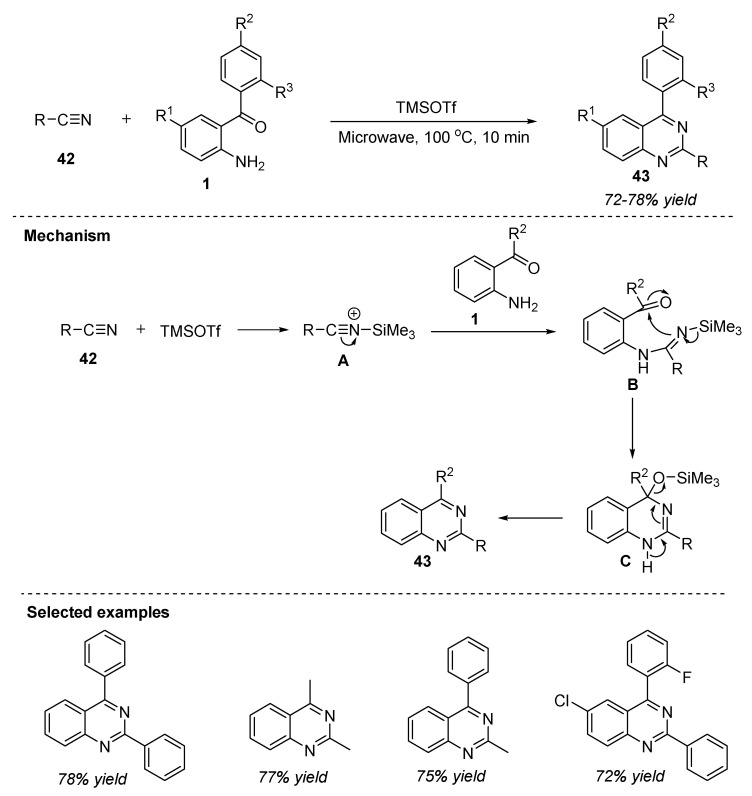
TMSOTf-catalyzed reaction for the synthesis of quinazolines.

In the recent years, reports have shown that the treatment of amines with nitriles in the presence of Lewis acids such as trimethylsilyl iodide (TMSI) led to the in situ generation of amidines [83]. In this regard, Pahari et al. (2018) disclosed the synthesis of 2,4-disubstituted quinazolines **43** in 72–78% yields by the reaction between nitriles **42** and 2-aminobenzophenone **1** in the presence of the TMSOTf catalyst under microwave irradiation at 100 °C for 10 min (Scheme 20) [84]. Nitriles **42** activated by the Lewis acid catalyst, TMSOTf, were used as the nitrogen source in this reaction. The significant features of this approach include: single-step, mild and solvent-free conditions, in situ generated amidines, broad substrate scope, one-pot, and shorter reaction times. The reaction mechanism as illustrated in Scheme 7 involves the activation of electron-rich nitriles **42** by coordination with TMSOTf. Later, the nucleophilic attack of amine **1** onto the activated nitrile **A** generated an amidine intermediate **B** which then attacks the carbonyl to deliver quinazoline **43** via aromatization.

**Scheme 21:**
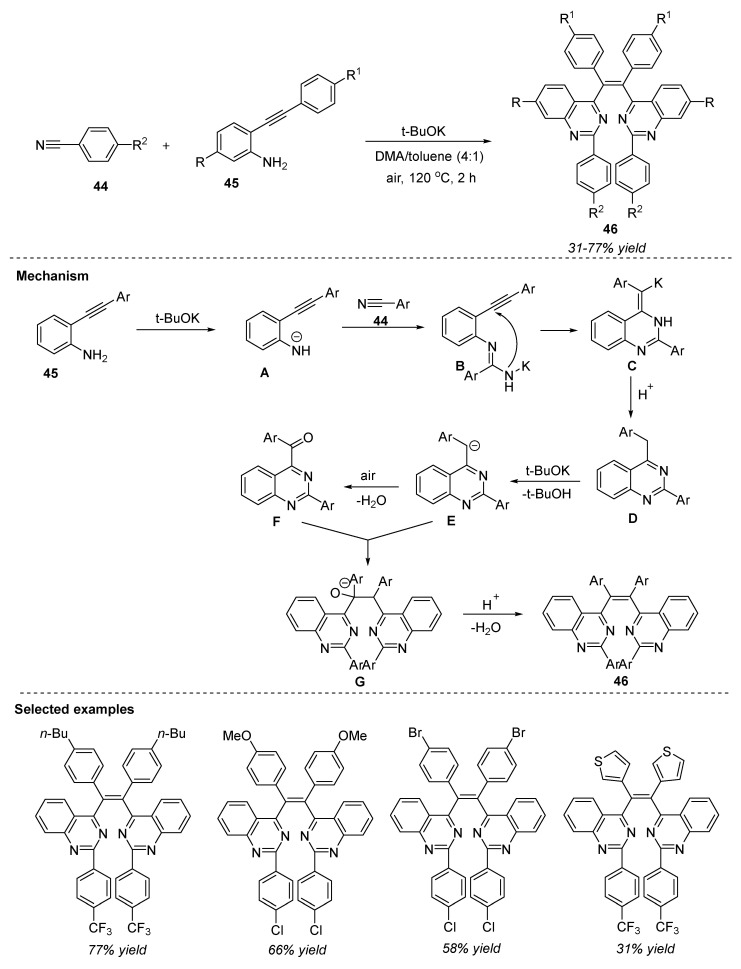
Metal-free chemoselective synthesis of π-conjugated quinazolines.

Conjugated π-systems with arenes or heteroarenes have gained attention due to their applicability in materials science. On the other hand, the synthesis of π-conjugated molecules under transition-metal-free conditions remains a challenge because of intrinsic mechanistic limitations [85]. In this connection, Jiang et al. (2018) developed a transition-metal-free chemoselective synthesis of π-conjugated quinazoline-substituted ethenes **46** by the reaction of 2-ethynylanilines **45** and benzonitriles **44** in the presence of KO^t^Bu and dimethylacetamide(DMA)/toluene (4:1) under air at 110 °C for 2 h (Scheme 21) [86]. Twenty-five derivatives were synthesized in moderate to high yields (31–77% yields). Interestingly, all the synthesized quinazolines **46** displayed a marked luminescence phenomenon. A variety of electron-donating and electron-withdrawing groups underwent the reaction and delivered the quinazolines **46** in a complete (*Z*)-configuration. Under optimized reaction conditions, unsubstituted and electron-donating-group-substituted benzonitriles **44** failed to give the desired product and this marks a major limitation in this approach.

The mechanism involved the initial deprotonation of 2-ethynylanilines **45** in the presence of a base to generate the anion intermediate **A**, which attacks the cyano group **44** selectively and leads to the formation of amidine intermediate **B**. Later, intermediate **C** is formed by the intramolecular cyclization of alkynes and amidines, followed by protonation, to result in intermediate **D**. Subsequent deprotonation, oxidation followed by the attack of **E** afforded **F**. In the last step, the desired product **46** is formed with the elimination of one water molecule from **G** (Scheme 21). The benzene ring at the 2-position of quinazoline drives the stereoselectivity by forming a stereoregular product.

**Scheme 22:**
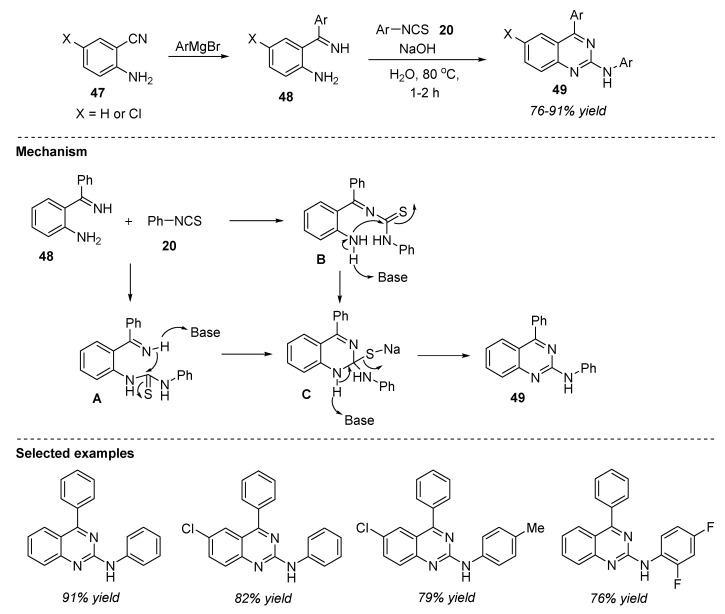
Base-catalyzed synthesis of quinazolines.

Palakodety et al. (2019) documented step economy and sustainable synthesis of substituted quinazolines **49** by utilizing the starting precursor 2-aminobenzonitrile **47**, Grignard reagent, and phenylisothiocyanate **20** in the presence of an inexpensive base NaOH and water as a solvent at 80 °C for 1–2 h [87]. The reaction of 2-aminobenzonitrile **47** with aryl magnesium bromide resulted in the formation of *ortho*-aminoketimine **48** [88]. Later, the reaction of *o*-aminoketimine **48** with isothiocyanate **20** and NaOH in water provided *N*,4-substituted quinazolines **49** in 76–91% yields (Scheme 22). The noteworthy advantages of this approach include: broad substrate scope, step economy, operational simplicity, shorter reaction times, good functional group tolerance, easy workup procedure, inexpensive precursors, and using water as an environmentally benign solvent.

The plausible mechanism detailed by the authors is depicted in Scheme 22. In the first step, *o*-aminoketimine **48** reacts with phenylisothiocyanate **20** to generate the thiourea intermediate **A**. In the next step, the subsequent intramolecular nucleophilic attack of the amine or imine group on the thiocarbonyl carbon led to the formation of the cyclized product **C** via N-C bond formation. Aromatization followed by subsequent liberation of S and H_2_O offered the desired quinazoline **49**.

## 3. Conclusions

Owing to the biologically active legacy, quinazolines have emerged as an important synthetic target in the last few decades. The transition-metal-free approaches in organic transformations are always more economical and complements metal-mediated reactions that suffer from drawbacks such as the possibility of toxic metal contamination in the final products, using expensive metal complexes, and the restricted applicability of products as pharmaceuticals, due to metal contamination. Consequently, rapid growth has been witnessed in the last few years in metal-free synthesis methods. Despite the efficacy of the reported protocols, they also suffer from inevitable disadvantages such as the use of excessive quantities of dangerous peroxide reagents, toxic solvents, toxic oxidants, longer reaction times, elevated temperatures, and requiring strong and hazardous oxidizing agents. Moreover, most of them are not solely focused on the development of an efficient approach with improved results viz., product yields, cost-effectiveness, atom-economy, and environmentally benign conditions. Hence, the development of novel synthetic strategies that proceed in a green reaction medium in the presence of benign reagents without the use of metal complexes, reagents, and hazardous organic solvents and also liberating minimum or benign waste are highly desirable.

This review highlights the recent progress achieved in the transition-metal-free synthesis of quinazolines and covers the literature from 2015 to 2022. It is presented alongside the advantages, limitations, and mechanistic rationalization associated with them. As discussed in the review, it is obvious that these strategies have noteworthy benefits such as readily available substrates, mild reaction conditions, broad substrate scopes, good to excellent yields, green conditions, gram-scale synthesis, etc. Furthermore, oxygen or air has been used instead of external toxic oxidants, and water as a green solvent made the strategies remarkable from a green and environmentally friendly point of view. In addition, employing microwave and visible-light irradiation techniques resulted in shorter reaction times and enhanced efficiency of reaction. Moreover, π-conjugated quinazolines displayed marked luminescence phenomena with excellent chemoselectivity. Furthermore, metal-free multicomponent reactions paved the way for the facile synthesis of substituted quinazolines. In spite of the merits achieved so far, some limitations such as prolonged reaction times, limited substrate scopes, and steric effects are also observed. Hence, metal-free methodologies should be developed in the near future, addressing these limitations which are efficient and environmentally benign. We believe the present review article will help researchers working on this fascinating area to construct quinazolines with immense applications in the field of medicinal chemistry.

## Data Availability

Not applicable.
